# Challenges in monitoring and titration of argatroban versus unfractionated heparin in extracorporeal membrane oxygenation

**DOI:** 10.1016/j.xjon.2026.102056

**Published:** 2026-08-18

**Authors:** Bernhard Nagler, Peter Schellongowski, Peter Quehenberger, Alexander Hermann, Mathias Schneeweiß-Gleixner, Elisabeth Lobmeyr, Michael Schwameis, Ludwig Traby, Nina Buchtele, Thomas Staudinger

**Affiliations:** a Department of Medicine I, Intensive Care Unit 13i2, Medical University of Vienna, Vienna, Austria; b Department of Laboratory Medicine, Medical University of Vienna, Vienna, Austria; c Division of Gastroenterology and Hepatology, Department of Medicine III, Intensive Care Unit 13H1, Medical University of Vienna, Vienna, Austria; d Department of Emergency Medicine, Medical University of Vienna, Vienna, Austria; e Division of Infectious Diseases and Tropical Medicine, Department of Medicine I, Medical University of Vienna, Vienna, Austria

**Keywords:** argatroban, heparin, extracorporeal membrane oxygenation, blood coagulation, thromboembolism

## Abstract

**Objectives:**

Anticoagulation during extracorporeal membrane oxygenation (ECMO) remains challenging. Direct thrombin inhibitors such as argatroban are increasingly proposed as alternatives to unfractionated heparin (UFH). We sought to characterize the dose titration and monitoring reliability of argatroban compared with UFH in adult patients who received ECMO.

**Methods:**

This prespecified subanalysis of a randomized controlled pilot trial (2021-2024) included 40 adults on venovenous or venoarterial ECMO randomized to argatroban (target diluted thrombin time 0.6-0.8 μg/mL; n = 20) or UFH (target anti-factor Xa 0.2-0.3 IU/mL; n = 20). The primary outcome was the pooled proportion of measurements within the therapeutic target range. Secondary outcomes included dose requirements, time to target, dose-assay correlations, and platelet count trajectories.

**Results:**

Patients who were treated with argatroban achieved therapeutic targets significantly less often than those receiving UFH (38% vs 51% of pooled active measurements; *P* < .001). The median argatroban dose required to reach the initial target was 0.40 μg/kg/min (interquartile range, 0.34-0.49). Correlations between anticoagulant dose and monitored effect were weak-to-moderate for both agents (repeated measures correlation coefficient = 0.29 for argatroban vs diluted thrombin time, and repeated measures correlation coefficient = 0.31 for UFH vs anti-Xa). Both groups experienced a comparable decrease in platelet counts.

**Conclusions:**

In this hypothesis-generating subanalysis, patients receiving argatroban achieved therapeutic targets less reliably than those receiving UFH. However, this variability likely reflects challenges in dosing strategy optimization and differing target stringencies rather than intrinsic pharmacokinetic instability of the drug.

**Figure:**
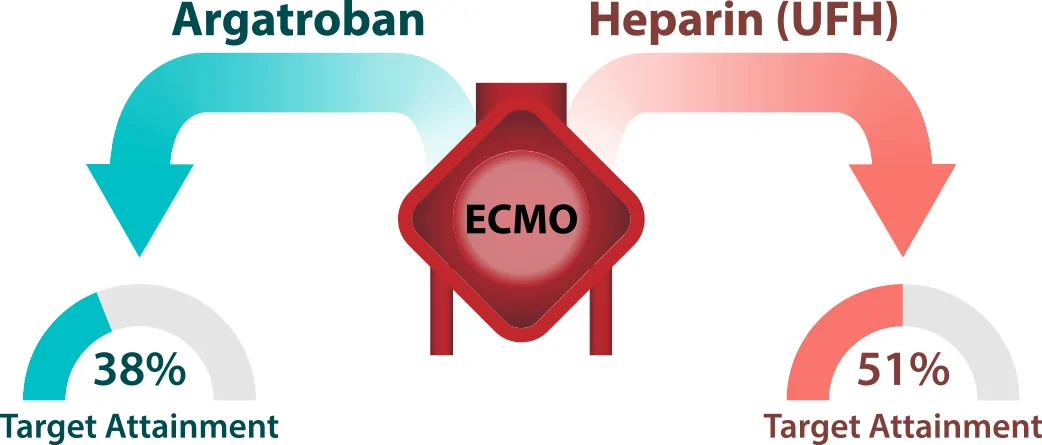
UFH achieved prespecified anticoagulation targets more often than argatroban during ECMO.

**Central Message**

Argatroban achieved therapeutic targets less reliably than UFH in patients on ECMO. Intensified starting doses and refined target ranges are required to match the monitoring stability of heparin.

**Perspective**

Argatroban achieved therapeutic targets less reliably than UFH in this ECMO cohort, primarily due to conservative initial dosing and stringent monitoring targets. These hypothesis-generating results highlight challenges in dosing optimization. Future research should refine target ranges and develop intensified starting protocols to improve argatroban's clinical controllability.

Optimal monitoring and dosing of anticoagulation during extracorporeal membrane oxygenation (ECMO) remains a major clinical challenge.^1^ Ideally, anticoagulant therapy should prevent thromboembolism and maintain circuit patency while minimizing bleeding risks. Whereas the most commonly used anticoagulant during ECMO is unfractionated heparin (UFH),^2^ several alternative agents have been proposed, including direct thrombin inhibitors (DTIs) such as bivalirudin or argatroban, which are theoretically less likely to induce platelet activation than heparin.^3^ Because of its dependence on antithrombin and ability to bind to several proteins, including acute-phase reactants,^4^ UFH dosing is challenging in patients who are critically ill, even though recent data suggest that acquired antithrombin deficiency during ECMO may be transient and of limited clinical relevance.^5^ For monitoring the effects of UFH, activated partial thromboplastin time (aPTT), or anti-factor Xa (anti-Xa) activity levels are used. DTIs, in contrast, act independently from other proteins by direct thrombin (factor IIa) inhibition, therefore suggesting more predictable effects.^1^ Proposed monitoring strategies for argatroban include aPTT and diluted thrombin time (dTT; such as the HEMOCLOT assay [HYPHEN BioMed]).^6^^,^^7^ In our recent randomized controlled pilot trial,^8^ we compared argatroban with UFH and found no difference in bleeding or thromboembolic events. Building upon those clinical safety findings, the present prespecified subanalysis focuses strictly on the pharmacologic management of these agents, aiming to characterize the dose titration and monitoring reliability of argatroban compared with UFH.

## Methods

### Study Design and Study Patients

This is a prespecified subanalysis of a prospective, randomized controlled pilot trial conducted at the Medical University of Vienna (2021-2024). The full methodology and primary results of the parent trial have been reported previously.^8^ To summarize, the primary end point of the parent trial focused on clinical outcomes (time free from complications, encompassing Bleeding Academic Research Consortium–defined bleeding and thromboembolic events), which showed no significant difference between the 2 study arms. The present subanalysis exclusively investigates the distinct laboratory and titration dynamics of the cohort. The study included 40 adult recipients of venovenous (VV) or venoarterial (VA) ECMO with an anticipated duration of more than 24 hours. Exclusion criteria were a history of heparin-induced thrombocytopenia (HIT), excessive bleeding risk or contraindications to anticoagulation, pregnancy, severe liver disease, postoperative admission, presence of a lupus anticoagulant, or acquired intrinsic clotting factor deficiency.

### Study Intervention and Titration Protocols

Participants were randomized in a 1:1 ratio to receive argatroban (n = 20) or UFH (n = 20) for anticoagulation, administered until ECMO removal or death. At the time of ECMO cannulation, patients received an intravenous UFH bolus of 50 to 100 IU/kg. In the argatroban group, continuous infusion was initiated at a dose of 0.2 μg/kg/min and titrated to a target dTT level of 0.6 to 0.8 μg/mL. Dose adjustments were strictly guided by a predefined, stepwise titration protocol detailed in Table E1. In summary, dose adaptations ranged from standard adjustments of ±0.1 to ±0.2 μg/kg/min on the basis of the degree of deviation from the target dTT, including a 1-hour pause for extreme supratherapeutic levels (>1.5 μg/mL). The UFH group received an initial infusion of 5 to 10 IU/kg/h, adjusted to an anti-Xa of 0.20 to 0.30 IU/mL. Because UFH is the established standard of care at our institution, dose adjustments were performed on the basis of clinician discretion and routine practice (typically in increments of 100-250 IU/h) rather than a rigid, stepwise titration nomogram. Anticoagulation targets were identical for both VV ECMO and VA ECMO. Patients on VV ECMO in the UFH arm routinely received additional prostaglandin E_1_ on the basis of the results of our previous study.^9^

### Rationale for Argatroban Anticoagulation Targets

Establishing appropriate target ranges for factor IIa–based monitoring remains challenging. The Swiss Society of Hematology recommends a dTT target of 0.4 to 1.5 μg/mL for patients with HIT,^10^ aligning with in vitro data correlating aPTT levels 1.5 to 3.0 times baseline to a dTT of 0.25 to 1.5 μg/mL.^11^ Considering available data in patients with HIT^12^ and emerging evidence supporting the use of lower-intensity anticoagulation during ECMO, we selected a dTT target of 0.6 to 0.8 μg/mL.

### Monitoring of Anticoagulation

Importantly, activated partial thromboplastin time (aPTT) was measured for observational purposes only and was not used to guide dose adjustments in either study arm. Coagulation studies were performed at least twice daily and exactly 4 hours after any anticoagulant dose adjustment. For argatroban, if dTT levels were stable within the target range (0.6-0.8 μg/mL), the next control was scheduled within 4 to 12 hours. Routine laboratory monitoring included aPTT (reference range, 27-41 seconds; STA-PTT Automate 5, Diagnostica Stago; reagent containing dextran sulfate without added antithrombin), anti-Xa (reference range, <0.1 IU/mL; coagulometric assay, reagent containing dextran sulfate; Diagnostica Stago), and–in patients receiving argatroban–an additional dTT assay (reference range, <0.1 μg/mL; HEMOCLOT Thrombin Inhibitor Assay; HYPHEN Biomed). The assay was calibrated using commercially available, argatroban-specific calibrators per the manufacturer's protocol, with internal validation performed routinely by our central laboratory.^13^ Blood samples were drawn from the arterial line into citrated tubes (3.2% sodium citrate) and processed within 1 hour. This trial was approved by the Ethics Committee of the Medical University of Vienna (reference number: 1278/2021, approved April 26, 2021) and was conducted in accordance with the latest version of the Declaration of Helsinki, following GCP guidelines. Because eligible patients were unable to provide informed consent before participation, consent was obtained as soon as they regained consciousness where possible, in accordance with Austrian law. This trial was registered with EudraCT (2021-001456-34) and clinicaltrials.gov (NCT05226442) before the recruitment of the first patient. Reporting follows Consolidated Standards of Reporting Trials guidelines.^14^

### Outcomes

Although the primary end point of the main trial evaluated clinical safety and efficacy (bleeding and thromboembolism),^8^ the present subanalysis focuses entirely on the pharmacologic titration and monitoring of the anticoagulants. Therefore, the primary end point for this secondary analysis was the pooled proportion of all coagulation assay measurements within the prespecified target range across the entire treatment group. Measurements obtained during intended anticoagulation pauses were excluded from the primary analysis to accurately reflect active therapeutic exposure. Secondary end points included the number of measurements and median doses required to reach the initial targets, overall dose requirements during ECMO therapy, the correlation between anticoagulant dose and corresponding coagulation studies, and the trajectory of daily platelet counts during the first 9 days of ECMO. Two sensitivity analyses were performed: (1) evaluating target attainment exclusively during the maintenance phase, defined as all measurements after the first therapeutic value, and (2) calculating the continuous time in therapeutic range using linear interpolation to account for variable laboratory sampling frequencies.

### Sample Size

No formal sample size calculation was performed because of the pilot nature of the study.

### Statistical Analysis

Data were categorized according to group assignment (intervention or control) for subsequent analysis. Categorical variables are presented as counts and percentages and were compared using χ^2^ tests or Fisher exact tests, as appropriate. Continuous variables are presented as median and 25%- 75% interquartile ranges (IQRs). Comparisons were made using independent samples *t* tests or Mann-Whitney *U* tests, respectively. For the primary end point of this subanalysis, the percentage of measurements within the therapeutic target range was calculated as the number of values within range divided by the total number of measurements for each cohort. The Pearson χ^2^ test was used to compare the pooled proportions of categorical target attainment.

To analyze dose requirements over time, daily median doses were calculated for surviving patients on ECMO. Due to the changing sample size over time (censoring due to weaning or death), corresponding longitudinal data are presented descriptively. The continuous time in target range (TTR) was calculated using the Rosendaal method of linear interpolation, to account for variable laboratory sampling frequencies.^15^ To properly account for within-subject variance and the lack of independence in serially sampled intensive care unit data, the repeated measures correlation coefficient (*r*_rm_) was used to evaluate the relationships between drug doses and coagulation test results.^16^ Locally estimated scatterplot smoothing was used to visualize these relationships. Correlation coefficients were predefined as weak (<0.3), moderate (0.3-0.5), or strong (>0.5). Because of the exploratory character of this pilot study, no correction for multiple testing was performed. Statistical analysis was performed using R (version 4.5.2; R Foundation for Statistical Computing).

## Results

### Study Population and Sampling

A total of 809 coagulation assays were analyzed from the 40 randomized patients, comprising 402 dTT tests in the argatroban group and 407 anti-Xa tests in the UFH group. Baseline characteristics were largely balanced between the 2 groups, although patients assigned to the argatroban arm were significantly older than those in the UFH arm (median 65 vs 58 years; *P* = .037) (Table 1).

**Table 1:** Baseline characteristics

Variable	Overall, N = 40∗	Argatroban, n = 20∗	UFH, n = 20∗	*P* value†
Age, y	59 (51, 66)	65 (57, 68)	58 (46, 63)	.037
Male sex	33 (83%)	15 (75%)	18 (90%)	.4
BMI, kg/m^2^	27.7 (24.8, 30.9)	28.5 (25.6, 31.1)	26.3 (24.8, 30.3)	.6
SAPS 3 score, points	68.0 (63.0, 72.0)	67.0 (60.5, 72.5)	68.5 (66.0, 72.0)	.5
ECMO configuration				.8
VA ECMO	19 (48%)	9 (45%)	10 (50%)	
VV ECMO	21 (53%)	11 (55%)	10 (50%)	
Indication for ECMO				>.9
ARDS	16 (40%)	8 (40%)	8 (40%)	
Hypercapnia	5 (13%)	3 (15%)	2 (10%)	
eCPR	19 (48%)	9 (45%)	10 (50%)	
Platelet count on ECMO before randomization, G/L	165 (134, 234)	173 (127, 225)	163 (140, 234)	.8

*UFH*, Unfractionated heparin; *BMI*, body mass index; *SAPS 3*, Simplified Acute Physiology Score III; *ECMO*, extracorporeal membrane oxygenation; *VA*, venoarterial; *VV*, venovenous; *ARDS*, acute respiratory distress syndrome; *eCPR*, extracorporeal cardiopulmonary resuscitation; *G/L*, giga per liter.

∗ Median (interquartile range); n (%), calculated within each column.

† Wilcoxon rank sum test; Fisher exact test; Pearson χ^2^ test.

### Target Attainment and Anticoagulation Control

Regarding the primary specific aim of this subanalysis, patients treated with argatroban achieved their therapeutic targets significantly less frequently than those receiving UFH. The percentage of measurements within the therapeutic range was 38% for argatroban compared with 51% for UFH (*P* < .001). The daily anticoagulant doses and monitoring test results are visualized in Figure 1. Analysis of deviations revealed that although the proportion of supratherapeutic measurements was similar between groups, the argatroban group exhibited a significantly greater proportion of subtherapeutic measurements compared with the UFH group (43% vs 32%; *P* < .001). The percentage of TTR was significantly lower in the argatroban group (38% [IQR, 0%- 45%] vs 48% [IQR, 22%- 65%], *P* = .046). In a sensitivity analysis restricted to measurements obtained after the initial therapeutic target was achieved, target attainment remained significantly lower in the argatroban group compared with the UFH group (135 [43%] vs 192 [55%]; *P* = .003). Furthermore, the argatroban cohort exhibited a significantly greater proportion of supratherapeutic measurements (70 [23%] vs 50 [14%]; *P* = .010).

**Figure 1:**
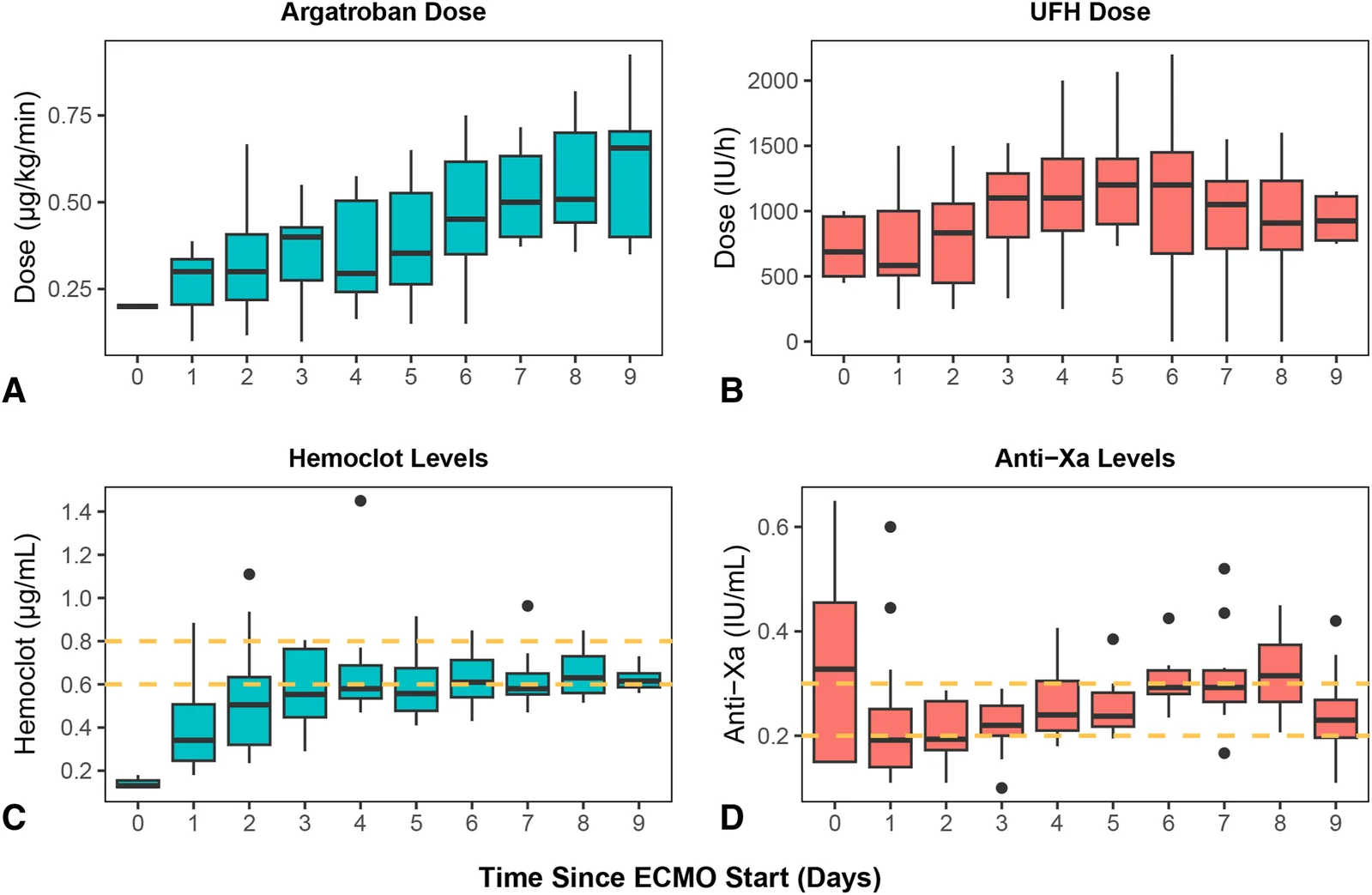
Dynamics of anticoagulation dosing and target attainment. Longitudinal analysis of daily anticoagulant doses and monitoring parameters over the first 9 days of ECMO support. A, Daily maintenance doses of argatroban (μg/kg/min), showing a progressive increase in dose requirements over time. B, Daily maintenance doses of UFH (IU/h). C, Monitoring of argatroban therapy using the specific dTT assay (μg/mL). D, Monitoring of UFH therapy using anti-Xa activity levels (IU/mL). The *horizontal dashed yellow lines* represent the protocol-defined therapeutic target ranges (dTT: 0.6-0.8 μg/mL; anti-Xa: 0.2-0.3 IU/mL). The *lower and upper borders of the colored boxes* represent the lower and upper quartiles (25th and 75th percentiles), respectively. The *middle horizontal black line* represents the median. The *whiskers* represent the minimum and maximum values of nonoutliers (within 1.5 times the IQR). *Extra dots* represent outliers. *ECMO*, Extracorporeal membrane oxygenation; *UFH*, unfractionated heparin; *dTT*, diluted thrombin time; *anti-Xa*, anti-factor Xa; *IQR*, interquartile range.

### Dosing Requirements and Dynamics

The median argatroban dose required to reach the initial therapeutic target (dTT, 0.6-0.8 μg/mL) was 0.40 μg/kg/min (IQR, 0.34-0.49), which was notably greater than the protocol-defined starting dose. Conversely, the median UFH dose required to reach anti-Xa targets for the first time was 750 IU/h (IQR, 500-1000), corresponding to approximately 7 IU/kg/h. The target level was reached after a median of 5 (IQR, 3-7) measurements in the argatroban group versus 3 (IQR, 2-4) in the UFH group (*P* = .033), whereas the time until reaching the target for the first time was not significantly different (33 [IQR, 22-45] vs 23 [17-30] hours, *P* = .072. The target was never achieved in 4 (20%) of patients receiving argatroban versus 2 (10%) of patients in the UFH group (*P* = .7). Longitudinally, argatroban dose requirements showed a progressive increase over the first 9 days of therapy, whereas UFH doses plateaued after day 5.

### Dose-Response Relationships and Assay Correlations

Correlations between the administered anticoagulant dose and the monitored effect were generally poor, reflecting the known physiologic variability in critically ill ECMO patients (Figure 2). The argatroban dose showed only a weak correlation with the specific dTT assay (*r*_rm_ = 0.29, *P* < .001) and a moderate correlation with aPTT (*r*_rm_ = 0.33, *P* < .001). In the UFH group, the prescribed dose demonstrated a strong positive correlation with aPTT (*r*_rm_ = 0.57, *P* < .001) and a moderate correlation with anti-Xa levels (*r*_rm_ = 0.31, *P* < .001). Regarding assay agreement, aPTT correlated strongly with anti-Xa in the UFH group (*r*_rm_ = 0.58, *P* < .001) and with dTT in the argatroban group (*r*_rm_ = 0.54, *P* < .001).

**Figure 2:**
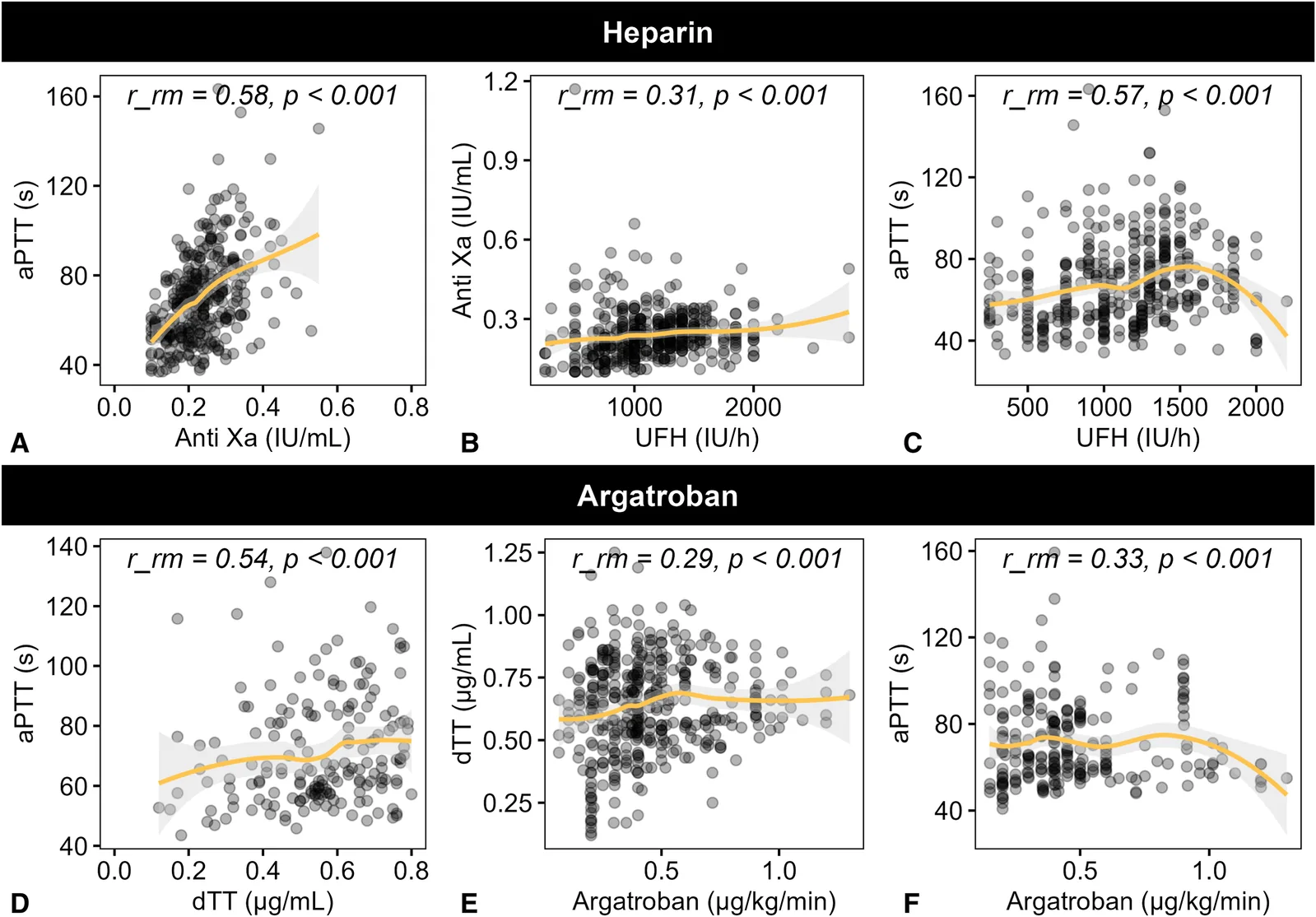
Correlations between anticoagulant doses and monitoring assays. Scatter plots with LOESS smoothing lines (*yellow*) and 95% CIs (*gray shading*) displaying the relationships between study drug doses and coagulation parameters, as well as the agreement between different coagulation assays. *LOESS*, Locally estimated scatterplot smoothing; *r*_*rm*_, repeated measures correlation coefficient; *aPTT*, activated partial thromboplastin time; *anti-Xa*, anti-factor Xa; *UFH*, unfractionated heparin; *dTT*, diluted thrombin time.

### Platelet Trajectories

The median platelet count of patients on ECMO before randomization was 165 G/L (IQR, 134-234 G/L), with no difference between the study groups. Patients had a median platelet decrease of 51% (42%- 67%) within a median of 3.6 days (IQR, 2.1-6.6 days) with no difference between groups. During the ECMO course, there was no difference in daily assessed platelet counts between patients receiving UFH and argatroban (Figure 3).

**Figure 3:**
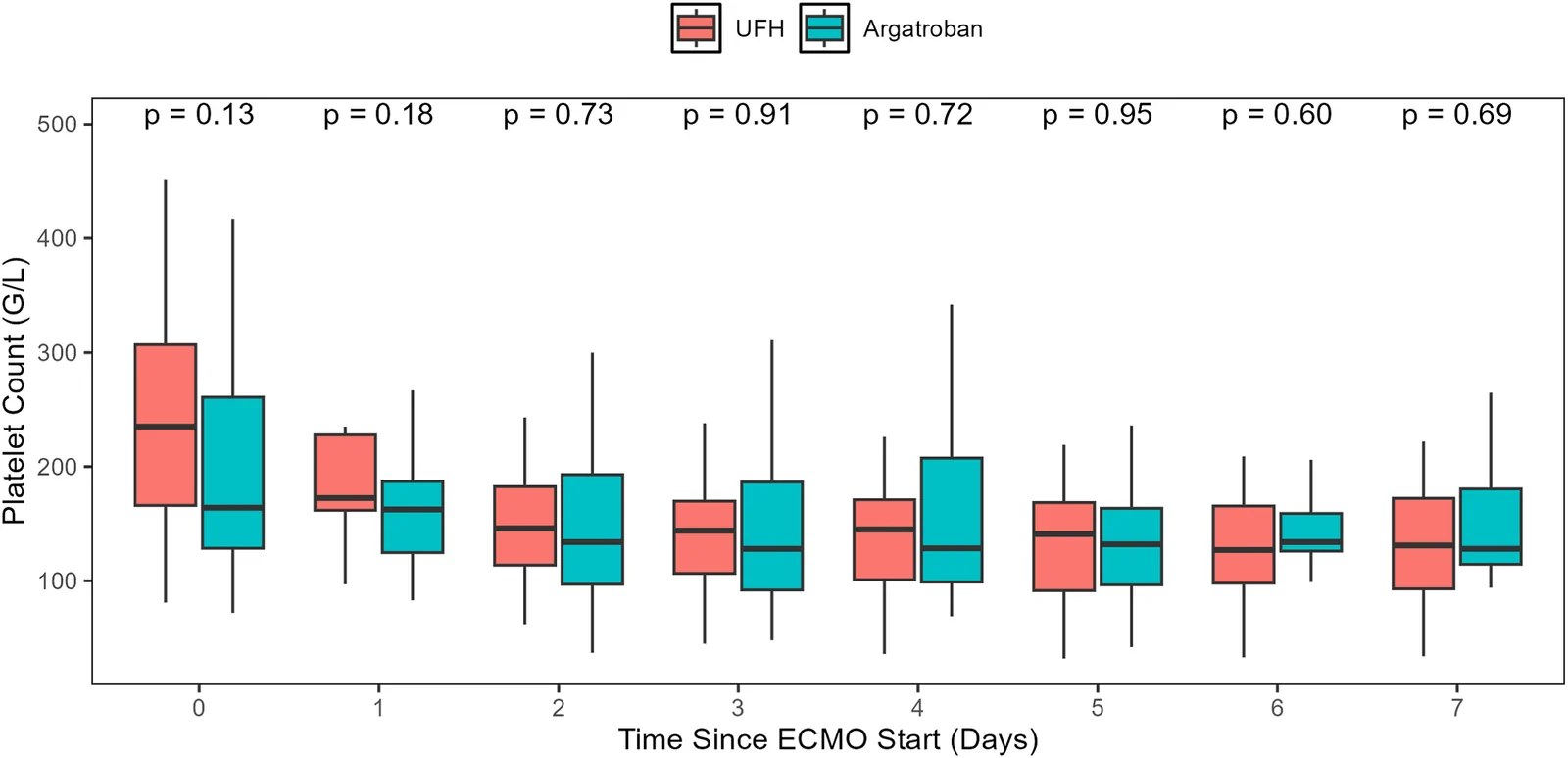
Platelet count trajectories. Longitudinal analysis of daily platelet counts (G/L) over the first 7 days of ECMO support. *Boxplots* represent the median (*horizontal black line*), interquartile range (IQR; *colored box*), and whiskers (1.5 × IQR). *Red boxes* represent the UFH group, and *blue boxes* represent the argatroban group. *P* values displayed at the top of the panel indicate daily between-group comparisons (Mann-Whitney *U* test), showing no statistically significant differences at any time point. *G/L*, Giga per liter; *ECMO*, extracorporeal membrane oxygenation; *UFH*, unfractionated heparin.

## Discussion

In this subanalysis of a randomized controlled pilot study, we aimed to evaluate the “predictability” of argatroban versus UFH in ECMO patients. Contrary to expectations, patients randomized to argatroban achieved therapeutic targets significantly less frequently than those receiving UFH.

### Anticoagulation Dosing and Monitoring

Our findings highlight the significant challenges of dosing strategy optimization during ECMO. The primary driver of lower target attainment in the argatroban group was a high prevalence of subtherapeutic measurements. The initial target was reached after significantly more measurements in the argatroban group, reflecting the conservative nature of the argatroban starting dose. Although our protocol used a starting dose of 0.2 μg/kg/min, the median dose required to achieve the therapeutic dTT target was 0.40 μg/kg/min. Although this is substantially lower than the standard 2.0 μg/kg/min used in patients with HIT who were noncritically ill,^17^ it exceeds maintenance doses previously reported in other ECMO and acute respiratory distress syndrome cohorts.^18^^,^^19^ This discrepancy implies that fixed, low-dose initiation protocols derived from previous studies may be insufficient for patients receiving ECMO who exhibit high thrombin generation potential, particularly in the context of COVID-19.^20^ Consequently, the target attainment variability observed here likely reflects a conservative initiation strategy rather than intrinsic pharmacokinetic instability of the drug. Future protocols targeting this intensity should likely consider greater starting doses to minimize time outside the therapeutic range. However, even after the initial target was achieved, target attainment remained poorer in the argatroban group, largely driven by a significantly greater rate of supratherapeutic measurements. This suggests that once therapeutic, argatroban levels were more difficult to keep from overshooting.

Furthermore, using repeated measures analysis to account for intrapatient variance revealed that dose-assay coupling was weak-to-moderate for both agents. The correlation between the administered dose and the specific monitoring assay was weak for argatroban and moderate for UFH. These findings underscore the substantial physiologic variability and altered pharmacokinetics inherent to patients who are critically ill on ECMO.^1^ Because of this unpredictable dose-response relationship, empiric dose adjustments determined by clinical weight alone are insufficient to achieve safety targets for either agent.

Interestingly, although aPTT is frequently confounded by acute-phase reactants, factor consumption, and baseline coagulopathy in this population,^21^ it demonstrated surprisingly robust performance in our repeated measures analysis. aPTT exhibited a moderate correlation with the argatroban dose and a strong correlation with the UFH dose while also maintaining strong agreement with both specific assays. Nevertheless, although aPTT retains utility as a widely available and rapidly resulting supplementary metric, we believe that using specific assays–despite the challenge of establishing perfectly equivalent target ranges–still appears to be a more attractive option to safely navigate the complex coagulopathies of ECMO.

Our findings align with recent pooled data from a systematic review and meta-analysis comparing DTIs with UFH specifically in patients supported by ECMO, which similarly highlighted the challenges of dose titration and target attainment associated with argatroban in this complex population.^22^

### Agent-Specific Versus Class Effects

Importantly, our findings regarding the weak dose-effect correlation of argatroban cannot be generalized to all DTIs, such as bivalirudin. The underlying randomized controlled pilot trial was strictly designed to only compare argatroban and UFH. Despite argatroban and bivalirudin both being DTIs, these agents possess fundamentally distinct pharmacologic profiles. Argatroban is a univalent DTI that binds exclusively to the active catalytic site of thrombin and relies entirely on hepatic metabolism via hydroxylation and aromatization.^23^ In the ECMO population, fluctuating hepatic function–frequently driven by venous congestion, altered systemic perfusion, or systemic inflammation–can lead to profound intra- and interpatient variability in argatroban clearance.^24^ This metabolic vulnerability likely contributes to the unpredictability and high prevalence of subtherapeutic measurements observed in our cohort. Conversely, bivalirudin is a bivalent DTI (binding both the active site and exosite-1) that is primarily cleared via intravascular proteolysis, with only ∼20% undergoing renal elimination.^25^ Because bivalirudin's enzymatic degradation is largely independent of hepatic organ function, it possesses a much shorter half-life (∼25 minutes) and theoretically more stable and predictable pharmacokinetics in critically ill patients. The aforementioned systematic review and meta-analysis^22^ demonstrated that patients receiving bivalirudin achieved therapeutic anticoagulation significantly faster and maintained target ranges for a greater proportion of time compared to those receiving UFH. Crucially, however, these dosing reliability benefits were not observed in the pooled subgroup of patients receiving argatroban. These pooled findings mirror the titration challenges observed in our cohort and stand in contrast to the superior target attainment of bivalirudin. Consequently, the inferior target attainment observed in this trial should be viewed as an argatroban-specific limitation rather than a broader failure of the DTI class.

### Impact on Platelets

Thrombocytopenia, or a decrease in platelet count, is common during ECMO and usually occurs within the first 3 to 7 days.^26^^,^^27^ In agreement with previous literature, we observed a platelet count decrease of approximately 50% by day 4. This decrease is likely multifactorial, with shear stress-induced platelet activation and consumption within the ECMO circuit being significant contributors.^27^ A previous report suggests that argatroban leads to a less-pronounced platelet decrease than UFH, possibly due to non-immunological HIT type I.^3^ However, we found no difference in daily platelet counts between the 2 groups, but this could also be explained by the small sample size with its obvious limitations.

A key limitation of the study is the lack of blinding, because different monitoring assays were used for each treatment arm. We cannot exclude that the difference in target attainment may stem from mismatched definitions of anticoagulation intensity. We targeted a dTT level of 0.6 to 0.8 μg/mL on the basis of HIT protocols, which likely represents a moderate-to-high anticoagulation intensity. In contrast, the UFH target (anti-Xa 0.2-0.3 IU/mL) represents a lower intensity strategy. Consequently, defining the optimal dTT target range for patients treated with ECMO should be a priority for future research. Second, the reliance on discrete laboratory assessments introduces a risk of sampling frequency bias; unstable patients with fluctuating coagulation profiles inherently undergo more frequent blood sampling than stable patients, potentially skewing the pooled sample estimate toward out-of-range values. To rigorously mitigate this limitation, we performed a continuous TTR sensitivity analysis using linear interpolation, which accounts for time exposure rather than discrete sampling events. This analysis confirmed our primary findings, demonstrating a significantly lower TTR in the argatroban cohort.

## Conclusions

In this hypothesis-generating subanalysis of a randomized controlled pilot trial, the specific argatroban protocol investigated resulted in lower therapeutic target attainment compared with standard UFH management, as evidenced by lower pooled target attainment and lower TTR. The prespecified argatroban protocol proved challenging to titrate, because the conservative starting dose required more frequent adjustments to achieve initial targets, whereas the maintenance phase was complicated by a greater propensity for supratherapeutic deviations. Furthermore, repeated measures analysis revealed that dose-response correlations for both agents were only weak-to-moderate, underscoring the profound physiologic variability inherent to ECMO. Consequently, the effective use of argatroban in ECMO highlights the need for dosing strategy optimization, requiring the development of intensified starting nomograms and refined target ranges to match the stability of standard UFH protocols.

### Declaration of Generative AI and AI-Assisted Technologies in the Writing Process

During the preparation of this work, the authors used generative AI (Google Gemini) in order to make grammar corrections and to improve readability. After using this tool/service, the authors reviewed and edited the content as needed and take full responsibility for the content of the publication.

## Conflict of Interest Statement

N.B. reported investigator-initiated research grant from Mitsubishi Tanabe and investigator-initiated research grant from CSL Behring. T.S. reported investigator-initiated research grant from Mitsubishi Tanabe; investigator-initiated research grant from CSL Behring; and speaker fees from Getinge and Xenios. P.S. reported speaker fees from Getinge and Fresenius Medical. A.H. reported speaker fees from Getinge. All other authors reported no conflicts of interest.

The *Journal* policy requires editors and reviewers to disclose conflicts of interest and to decline handling or reviewing manuscripts for which they may have a conflict of interest. The editors and reviewers of this article have no conflicts of interest.

## Figures and Tables

**Table E1:** Argatroban titration protocol

dTT	Dose adaptation	Next control
<0.25	+0.2 μg/kg/min	4 h
0.25-0.6	+0.1 μg/kg/min	4 h
**0.6-0.8**	**None**	**4-12 h**
0.8-1.0	–0.1 μg/kg/min	4 h
1.1-1.5	–0.2 μg/kg/min	4 h
>1.5	1-h pause and –0.2 μg/kg/min	4 h

Initial dose: continuous infusion 0.2 μg/kg/min. Target dTT: 0.6-0.8 μg/ml. Bold formatting highlights the target range and corresponding management. *dTT*, Diluted thrombin time.
